# Cognitive-behavioral and dietary weight loss intervention in adult kidney transplant recipients with overweight and obesity: Results of a pilot RCT study (Adi-KTx)

**DOI:** 10.3389/fpsyt.2023.1071705

**Published:** 2023-04-11

**Authors:** Dana Coco Barchfeld, Ricarda-Katharina Vagi, Katrin Lüdtke, Elisabeth Schieffer, Faikah Güler, Gunilla Einecke, Burkard Jäger, Martina de Zwaan, Mariel Nöhre

**Affiliations:** ^1^Department of Psychosomatic Medicine and Psychotherapy, Hannover Medical School, Hannover, Germany; ^2^Department of Cardiology, Angiology and Critical Care Medicine, Philipps University, Marburg, Germany; ^3^Department of Sports Medicine, Hannover Medical School, Hannover, Germany; ^4^Department of Nephrology and Rheumatology, University Medical Center Goettingen, Goettingen, Germany; ^5^Department of Nephrology and Hypertension, Hannover Medical School, Hannover, Germany

**Keywords:** obesity, overweight, kidney transplant recipient, weight loss, renal transplant recipient, cognitive-behavioral intervention

## Abstract

The obesity epidemic and its health consequences have not spared the population of kidney transplant (KTx) candidates and recipients. In addition, KTx recipients are susceptible to weight gain after transplantation. Overweight and obesity after KTx are strongly associated with adverse outcomes. Therefore, we designed a randomized controlled, mono-center study to specifically test the effectiveness of a primarily cognitive-behavioral approach supplemented by nutritional counseling for weight reduction following KTx as the intervention group (IG) in comparison to a brief self-guided intervention as control group (CG). The study was registered in the German Clinical Trials Register (DRKS-ID: DRKS00017226). Fifty-six KTx patients with a BMI from 27 to 40 kg/m^2^ were included in this study and randomized to the IG or CG. Main outcome was the number of participants achieving a 5% weight loss during the treatment phase. Additionally, participants were assessed 6 and 12 months after the end of the 6-month treatment phase. Participants significantly lost weight without group differences. 32.0% (*n* = 8) of the patients in the IG and 16.7% (*n* = 4) of the patients in the CG achieved a weight loss of 5% or more. Weight loss was largely maintained during follow-up. Retention and acceptance rate in the IG was high, with 25 (out of 28) patients completing all 12 sessions and one patient completing 11 sessions. Short-term, cognitive-behaviorally oriented weight loss treatment seems to be feasible and acceptable for patients after KTx who suffer from overweight or obesity. This clinical trial was ongoing at the onset of the COVID-19 pandemic which might have influenced study conduct and results.

**Clinical Trial Registration**: https://clinicaltrials.gov/ DRKS-ID: DRKS00017226.

## 1. Introduction

Overweight and obesity have become major global health problems. In the German general population, about 20% of the adult population is obese (BMI ≥ 30 kg/m^2^) (1, 2). The obesity epidemic and its health consequences have not spared the population of kidney transplant candidates and recipients (3). Kidney transplantation (KTx) is the treatment of choice for patients with end-stage renal disease (4). Due to organ shortage, KTx candidates in Germany must wait on average 8 years before receiving a post-mortal organ donation. The percentage of patients receiving a living kidney donation was 23.8% in 2021 and decreased during the last years (5). Thus, many patients are on dialysis for several years before transplantation. During dialysis treatment, a higher BMI is associated with better survival (6–9). This phenomenon has been referred to as the obesity paradox. After KTx; however, overweight and obesity are associated with less favorable outcomes. Most research focuses on the association between obesity and post-transplant outcomes and comorbidities. Several meta-analyzes summarized the literature on the impact of obesity on patient outcomes after kidney transplantation (6, 10–13). They reported higher rates of complications early after transplantation, including delayed graft functioning and surgical complications as well as a higher rate of adverse long-term complications such as cardiovascular diseases. Regarding the association between obesity and graft loss as well as morbidity, the findings are controversial (6, 10–13). Undeniable is the association between obesity and higher rates of hypertension, dyslipidemia, and diabetes mellitus in kidney transplant recipients (14). Additionally, an association between overweight and the development of new-onset diabetes after transplantation (NODAT) and hypertension has been described (12). Overall, the BMI after KTx seems to be more strongly related to adverse long-term outcomes than the pre-transplant BMI (15).

There is evidence that KTx recipients are susceptible to weight gain after transplantation (16, 17). In a large German sample of patients before and after kidney transplantation (*n* = 433), the frequencies of obesity (BMI > 30 kg/m^2^) were 14.8% (before KTx) and 19.9% (after KTx), respectively. There was a strong association between post-transplant BMI categories and type 2 diabetes mellitus as well as new-onset diabetes after transplantation (NODAT). Above that, an association between increasing BMI and decreasing graft functioning (eGFR) was found (18). These results are cause for concerns and underline the necessity of obesity management in KTx patients. Multiple factors have been associated with post-transplant weight gain including relaxation of dietary restrictions, increased appetite and well-being, immunosuppressive medication (i.e., steroids), insulin treatment due to NODAT (19), and inadequate physical activity.

Even though overweight and obesity after KTx are strongly associated with adverse outcomes in the long run, there is still a lack of evidence from randomized controlled weight reduction trials. Overweight and obesity after transplantation are potentially modifiable risk factors for poor outcome and thus an appropriate target for therapeutic interventions. In a recent Cochrane review of Conley et al. (20) focusing on interventions for weight loss in patients with chronic kidney disease, only four studies included KTx patients. Orazio et al. (21) performed a mixed lifestyle intervention including dietary modifications and an increase of physical activity. Tomlinson et al. (22) evaluated the effects of an appetite suppressant compared to a dietary intervention. Tzvetanov et al. (23) investigated the effectiveness of a combined robotic KTx and sleeve gastrectomy versus robotic KTx and a conservative weight loss program. The authors came to the conclusion that lifestyle-based weight loss interventions may result in weight loss in KTx recipients. Due to the limited study situation, more specific recommendations could not be made.

There have been different intervention studies aiming at preventing or minimizing weight gain after KTx. Henggeler et al. (24) designed a randomized controlled trial comparing an intensive nutritional intervention with the goal to prevent weight gain during the first year after kidney transplantation with standard nutritional care. The mean weight increase over 12 months was 4.6% without any differences between the intervention and control group. In a feasibility study of Gibson et al. (25) focusing on a televideo physical activity and nutrition program, 10 KTx patients were randomly assigned to an intervention or a control group. Even though treatment adherence was high, the authors reported somewhat more weight gain in the intervention group compared to the control group. Apart from that, there are two RCTs in KTx recipients currently ongoing focusing on the prevention of weight gain (26) and the improvement of abnormal glucose metabolism parameters with weight changes as a secondary outcome (27).

Based on these results, it can be summarized that studies and study protocols published so far focus primarily on nutrition and exercise interventions to prevent or reduce weight gain or achieve weight loss in KTx patients. Studies concentrating on cognitive-behavioral health approaches as the primary mode of action have–to our knowledge–not been published so far. According to the German S3 guideline “Obesity–Prevention and Treatment” (28), an intervention for weight loss should include different aspects: diet, exercise, and cognitive-behavioral elements. Taking into account that KTx recipients, especially those who received dialysis treatment over several years, have a high level of comorbidity and are often physically impaired, it becomes obvious that a large proportion of the patients is not able to perform high level physical exercise as a weight reduction strategy (29). Therefore, an intervention with the aim to reduce weight specifically designed for KTx recipients should concentrate on the dietary and cognitive-behavioral aspects in addition to routine daily physical activity. Therefore, we designed a randomized controlled, mono-center study to specifically test the effectiveness of a primarily cognitive-behavioral approach supplemented by one session of nutritional and exercise counseling for weight reduction following KTx. A self-guided leaflet-based intervention focusing on habit formation (the Ten Top Tips, 10TT, 30) was chosen as a minimal intervention control group.

## 2. Methods

### 2.1. Participants

Between June 2019 and September 2020, 56 kidney transplant recipients interested in weight loss treatment were recruited at Hannover Medical School. Eligibility criteria included age ≥ 18 years, BMI 27 to 40 kg/m^2^, and ≥ 3 months after KTx. Additionally, participants needed sufficient German speaking skills and had to be able to give written informed consent. Exclusion criteria included severe cognitive impairments (DemTect ≤8; 31) and an unstable transplant function. An unstable graft function was defined as an acute rejection episode or rejection treatment within the last 3 months, urosepsis, acute cytomegalovirus infection or BK polyomavirus infection, or a decline of estimated glomerular filtration rate (eGFR) of 20 or more ml/min/1.73 m^2^ during the last 3 months. Patients who were currently pregnant or planned pregnancy soon were excluded as well. Special consideration was given to patients with cystic kidney disease. Based on the information from ultrasound scans on the size of the patients’ kidneys, only patients were included in whom merely the size of the polycystic kidneys did not explain the overweight.

**Figure 1:**
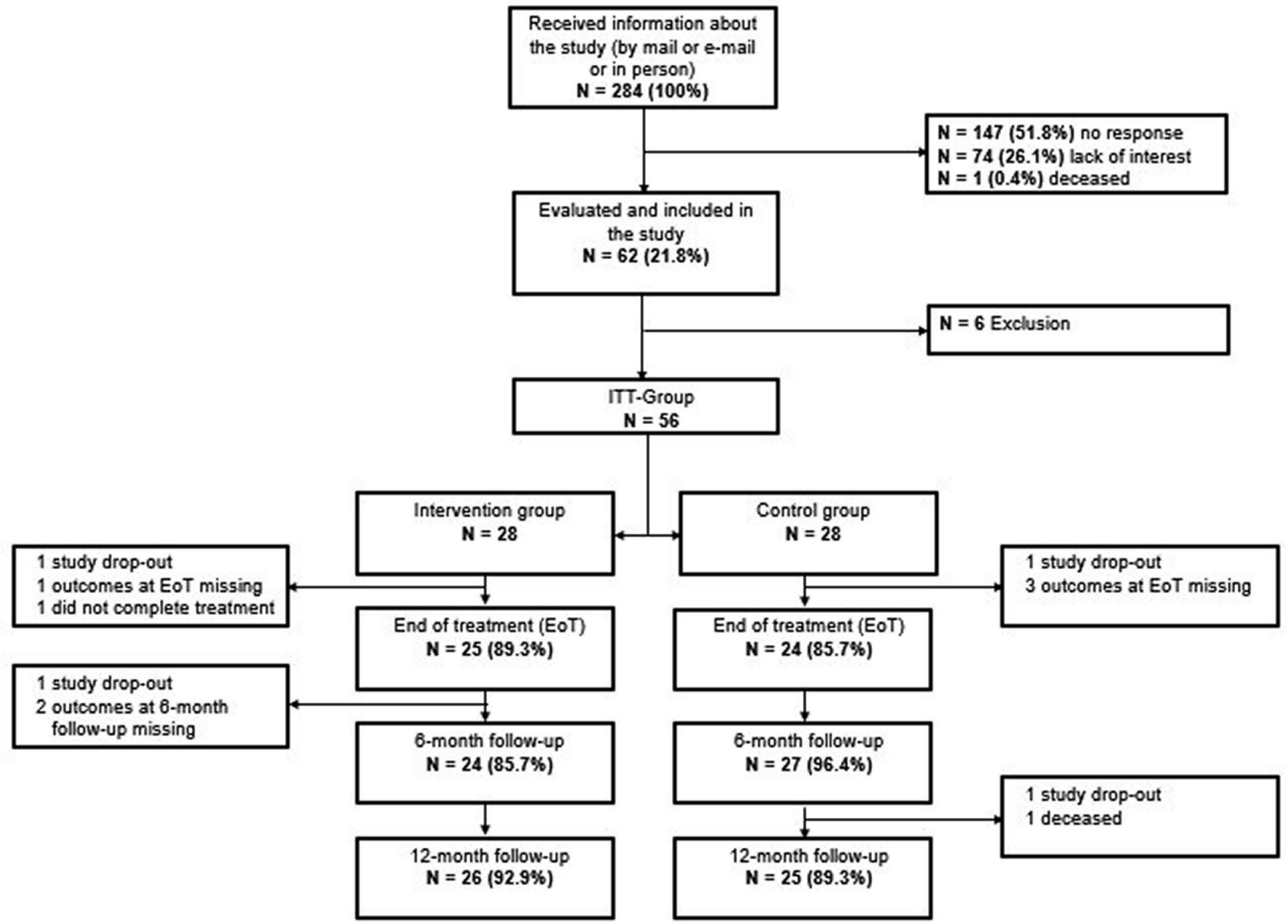
Patient flow.

Further details of the recruitment process can be found in Figure 1. Overall, 284 KTx recipients were informed about the study. As described in Figures 1, 62 patients were interested in the trial, while 74 actively declined participation and 147 did not respond to the information they received. Individuals who met the inclusion criteria and gave written informed consent were randomized. Of those, 6 were excluded as they did not fit the inclusion criteria at baseline visit (BMI below 27 kg/m^2^ or above 40 kg/m^2^, medical complications), did not show up for the baseline visit, or decided against study participation before participating in at least one post-baseline cognitive-behavioral assessment (IG) or the control intervention (CG). Fifty-six patients were successfully included in the study. They received at least one post-baseline assessment and constituted the intention-to-treat (ITT) analysis group.

The study was performed according to the Declaration of Helsinki. All participants gave written informed consent. The Institutional Ethics Review Board of Hannover Medical School approved the study (8341_BO_S_2019). The study was registered in the German Clinical Trials Register (DRKS-ID: DRKS00017226) on April 30^th^ 2019.

### 2.2. Primary outcome

The primary outcome was the percentage of participants achieving a weight loss of ≥5% at the end of the 6-month intervention period. A weight loss of ≥5% is considered to be a realistic and sustainable weight loss goal which is sufficient to significantly improve metabolic and immunological parameters according to the German guideline “Obesity–Prevention and Treatment” (28). Weight was measured at the beginning and end of treatment (6 months) using calibrated electronic scales and was recorded to the nearest 0.1 kg. Participants were weighed in light clothing and without shoes. Information on patient’s height were taken from the patient’s charts. Weight and height were used to calculate BMI.

### 2.3. Secondary outcomes

Secondary outcomes were changes in BMI, renal function parameters, quality of life, and levels of depression and anxiety. Additionally, longer-term effects 6 and 12 months after treatment completion were examined.

#### 2.3.1. Impact of weight on quality of life questionnaire-Lite

The Impact of Weight on Quality of Life Questionnaire (IWQOL-Lite; 32) is a weight-specific questionnaire to evaluate health-related quality of life (HRQoL). The instrument consists of 31 items focusing on concerns of individuals with overweight or obesity. Each item is rated on a 5-point Likert scale ranging from “never true” to “always true,” leading to a total score and five subscale scores (Physical Function, Self-Esteem, Sexual Life, Public Distress, and Work) (not reported in this paper). Lower scores indicate less impairment and better quality of life. For this study, the total score of the validated German version by Mueller et al. (33) was used.

#### 2.3.2. SF-12

The SF-12 is a validated instrument to evaluate generic health-related quality of life (HRQoL) (34). It consists of 12 questions resulting in two summary scores for physical (PCS-12) and mental (MCS-12) health. Scores are transformed and computed as *t*-scores with a mean of 50 and a standard deviation of 10, based on data from the US general population with the higher score indicating better HRQoL.

#### 2.3.3. Hospital anxiety and depression scale

Symptoms of anxiety and depression were measured using the German version of the Hospital Anxiety and Depression Scale (HADS) (35, 36). The validated self-report instrument was designed to assess levels of anxiety and depression specifically in patients with somatic comorbidities. The two subscales “depression” and “anxiety” consist of seven items each. Each item is scored from 0 to 3, leading to a total score between 0 and 21. Higher scores indicate higher levels of depression or anxiety.

### 2.4. Assessments

Assessments were performed at baseline, at the end of treatment (6 months after baseline), and 6 as well as 12 months after the end of treatment. Weight was measured and participants were asked to complete the IWQoL-Lite, the SF-12, and the HADS questionnaire.

### 2.5. Sociodemographic, transplant-specific, and medical parameters

Information on medical parameters such as estimated glomerular filtration rate (eGFR), diabetes mellitus and treatment of diabetes mellitus, other comorbidities, medication, and hospital stays during the study period were taken from the medical records. Participants who did not visit the transplant outpatient clinic due to restrictions during the Covid-19 pandemic were asked to send the latest doctor’s letter and laboratory values from their local nephrologist.

Sociodemographic and transplant-specific variables including sex, age, and partnership status, level of education, donation type, and time since KTx were assessed using a self-report questionnaire. Missing information was obtained from the medical records.

### 2.6. Intervention group

Since patients after KTx are a vulnerable group with a high proportion of comorbidities and an already complex medical regimen, we developed a primarily cognitive-behavioral intervention with 12 individual sessions over a period of 6 months following a manualized protocol. The intervention consisted of 12 individual sessions of 50 min each and was based on the already established “Lighter through Life” (“Leichter durchs Leben”) intervention, which is conducted at MHH by the Departments of Psychosomatic Medicine and Psychotherapy, of Gastroenterology, Hepatology, and Endocrinology and of Rehabilitation Medicine. The “Lighter through Life” program was accepted by all national and local relevant health insurance companies. In addition, the intervention included elements of the treatment manual “Binge Eating and Obesity” (37). Interventions that take account of the psychological processes underpinning eating behavior and focus on habitual behaviors may have greater long-term success than nutritional only interventions (38). As kidney transplant patients present a heterogeneous group with unique challenges, e.g., different comorbidities, the intervention in this study was performed in individual sessions. Sessions were offered face-to-face or telemedically (*via* video conferencing or telephone). This form of patient communication has been tested successfully in an aftercare program for KTx patients (KTx 360°; 39). As only a small proportion of the patients live close to the transplant center in Hannover, this approach might facilitate implementation and increase scalability of the intervention. Eleven of the 12 planned sessions were performed by a physician or a clinical psychologist from the Department of Psychosomatic Medicine and Psychotherapy. The intervention included cognitive-behavioral as well as psychoeducational elements. An overview of the content of the individual sessions is summarized in Table 1.

**Table 1:** Intervention modules.

Module	Contents
Module 1 getting started	Getting to know each other, weight/diet/eating history and goal setting
	Program overview
	Homework: Food diary
Module 2 nutritional and exercise counseling	Individualized nutritional counseling based on the food diaries and on the clinical condition after KTx
	Physical activity planning
Module 3 overweight and obesity	Exploring the course of body weight (weight graph) and eating “types”
	Education about obesity
	Homework: Food diary
Module 4 reasons for and against losing weight	Identifying individual reasons for and against losing weight
	Identifying weight loss barriers
	Homework: Motivational Strategies worksheet
Module 5 eating cues	Recognizing internal and external eating cues
	Using change strategies
	Optional: Exposure-based intervention with high-risk food
	Homework: Experimenting with different change strategies
Module 6 progress report	Progress report:
	➢Discussing progress in dietary change
	➢Defining goals for the upcoming weeks
	Homework: Food diary
Module 7 resources and behavioral change	Identifying resources and strengthening self-esteem
	Rearranging cues, changing responses to cues, rearranging consequences, changing thoughts
	Homework: Response-cues-consequences worksheet
Module 8 mindfulness	Promote enjoyment of food by mindfulness
	Mindful raisin exercise
	Homework: Behavioral change worksheet
Module 9 vicious circle	Understanding the thought-behavior connection leading to a vicious circle
	Identifying individual thought-behavior connection leading to a vicious circle
Module 10 stress management	How to deal with stress?
	List of pleasant activities
	Homework: Stress reduction worksheet
Module 11 repetition	Repetition of contents based on participant’s preference
	Homework: Maintenance plan worksheet
Module 12 relapse prevention	Recognize your progress; praise your accomplishments
	Strategies for relapse prevention
	Establishing an emergency plan

One session was performed by a physician specialized in nutritional medicine from the Department of Sports Medicine, with knowledge about the specific nutritional needs and exercise capacity of patients after KTx. Patients after KTx have special needs regarding germ-reduced nutrition, electrolytes, or proteins, making nutrition counseling more complex. Patients in the IG were asked to document their nutrition with a self-written food diary and photos over the course of 3 days. The recommendations were individualized and took into account personal preferences and abilities like cooking skills. In general, patients were counseled to follow a balanced, primarily Mediterranean diet. They were advised to consume more healthy components like vegetables, if the consumption was estimated to be too low. In addition, patients were asked to avoid the unhealthiest nutrients in their food diary and generally to limit the portions. The dietary intervention did not include a predefined calorie reduction.

Even though the study did not include active physical activity elements, patients were encouraged to continue their exercise routines and daily activities. Thus, we did not exclude patients who were unable to perform moderate to vigorous physical activity (PA) as in other studies that included active PA in their program (25).

### 2.7. Control group

The participants in the CG received a brief, self-guided intervention consisting of a leaflet containing healthy nutrition and activity recommendations and simple advice on habit formation (the Ten Top Tips, 10TT) (30). The 10TT is a single-session intervention with no further contact with a health professional, which was intended as a partial “control-for-attention.” The 10TT leaflet has shown to result in modest but significant weight loss in motivated volunteers with obesity but also in more heterogeneous primary care samples (40, 41). Thus, we did expect some weight loss also in this group.

### 2.8. Randomization

Participants were assigned randomly to the intervention group (IG) and the control group (CG), with 28 in each group. Randomization was performed using a randomization list. Randomization was supported by scientists who were not part of the study team.

### 2.9. Sample size estimation

The primary goal was to achieve a weight loss of ≥5% of baseline weight, which is the benchmark of successful and healthful weight loss. Sample size estimation for the primary outcome was based on findings of other trials using low intensity behaviorally oriented interventions (IG) and brief, self-guided interventions (CG) (40). We assumed that in the cognitive-behaviorally-oriented IG, 55% of participants would attain a ≥ 5% weight loss, while in the brief, self-guided CG, 15% would reach this goal. Thus, we expected a difference of 40% between groups. Power analysis yielded a target sample of 56 patients with 28 patients in each group to achieve a 90% power for the main outcome (40% difference in response criterion) at two-sided alpha level of 0.05. A treatment dropout was defined as a participant who completed less than half (less than six) of the therapy sessions. As renal transplant recipients are required to keep regular appointments at the transplant center, we did not expect study dropouts concerning the main weight outcome.

### 2.10. Statistical analysis

Analyzes were performed using IBM® SPSS® version 26.0 (IBM Corp.). Descriptive data are presented as *means* with standard deviation (SD) or numbers (*n*) and percentages.

As the primary analysis, a responder analysis was carried out comparing the number of patients successfully attaining a 5% weight loss or more between the IG and the CG using Chi-square (*χ*^2^) analysis. A completer (data at end of treatment available and attendance of at least 6 treatment sessions) and an ITT analysis including all patients from the ITT group (last observation carried forward, LOCF) were carried out. Phi (*φ*) (χ2-test) was reported as effect size (small: *φ* < 0.2; moderate: 0.25 < *φ* < 0.7; large: *φ* > 0.7). The sole use and often dichotomous interpretation of the *p* value has been frequently scrutinized in recent years. To prevent non-significant results from being falsely diminished in their relevance, several authors recommend concentrating on and reporting effect sizes, since they do not depend on factors such as sample size (42, 43).

Univariate statistical analysis comparing patients who reached and did not reach the 5% weight loss goal on sex, age, BMI at baseline, treatment with insulin, time since transplantation, and hospital stays was conducted using Chi-square analyzes and Mann–Whitney-U-tests as appropriate.

In a secondary analysis, changes in BMI were compared between the IG and CG using linear mixed models for repeated measures (MMRM). BMI at baseline, treatment group, and visit were included as fixed effects. To estimate how the change over time depends on the treatment group, a treatment-by-visit interaction was added as an additional fixed effect term. Participant (patient ID) was included as a random effect (random intercept). Three MMRMs were computed i.e., (1) data from baseline to the end of treatment (main outcome), (2) all data from baseline to 6-month follow-up, and (3) all data from baseline to 12-month follow-up. A restricted maximum likelihood (REML)-based approach was used to obtain estimates. Significance tests were based on estimated marginal means (LS means). The significance level for α was set at *p* < 0.05 for group differences.

Secondary analyzes included the analyzes of further outcome variables assessing quality of life (IWQOL-Lite total score, SF-12 composite scale scores), levels of anxiety and depression (HADS), and eGFR as a measure of kidney functioning. The analyzes were carried out using MMRM including all data from baseline to 12-month follow-up, with the secondary outcome variable as the dependent variable, and treatment group, visit, treatment-by-visit interaction, and the respective baseline value as fixed effects and participant as a random effect.

## 3. Results

### 3.1. Participants

Figure 1 shows the participant flow through the study. Overall, 56 patients were analyzed, 28 in each group. Treatment groups did not differ significantly in treatment completion rates (with one study drop out in each group) or assessment rates at post-treatment (IG 92.9%, CG 85.7%). Overall, 91.1% (*n* = 51) of the participants were assessed at 12-month follow-up; assessment rate did not differ by group (IG 92.9%, CG 89.3%).

In the IG, 25 completed all 12 sessions and one participant completed 11 sessions.

Table 2 summarizes the baseline characteristics. Participants had a mean age of 48 (SD 12.3) years, 27 participants (48.2%) were female, mean time since transplantation was 77.4 (SD 68.4) months with a wide range from 4 months to 30 years, and 19 (33.9%) were living donor recipients. Mean eGFR was 44.9 (SD 15.8) ml/min/1.73 m^2^, the majority were diagnosed with hypertension (83.9%), and about one-third (28.6%) suffered from diabetes mellitus. The mean BMI at baseline was 32.0 (SD 3.0), 32.1% (*n* = 18) were overweight, and the remaining participants were obese. Comparison between groups can be found in Supplementary Table S1.

**Table 2:** Participant characteristics at baseline.

*N*	Total	Intervention group (IG)	Control group (CG)
	56	28	28
**Age at baseline, years** Mean (SD)	48.0 (12.3)	48.2 (11.4)	47.7 (13.3)
**Sex, women** % (*n*)	48.2 (27)	42.9 (12)	53.6 (15)
**Educational level** ≥12 years of formal education, % (*n*)	26.8 (15)	28.6 (8)	25.0 (7)
**eGFR ml/min/1.73 m**^**2**^Mean (SD)	44.9 (15.8)	40.5 (14.1)	49.4 (16.5)
**Time since KTx, months** Mean (SD)	77.4 (68.4)	67.8 (69.8)	87.1 (66.9)
**Dialysis before KTx**, % (*n*)	80.4 (45)	89.3 (25)	71.4 (20)
**Living kidney donation**, % (*n*)	33.9 (19)	32.1 (9)	35.7 (10)
**Diabetes mellitus**, % (*n*)	28.6 (16)	28.6 (8)	28.6 (8)
Type 1, *n*	5	2	3
Type 2, *n*	6	4	2
NODAT, *n*	5	2	3
Insulin therapy, *n*	11	6	5
**Coronary heart disease** % (*n*)	8.9 (5)	10.7 (3)	7.1 (2)
**Hypertension** % (*n*)	83.9 (47)	85.7 (24)	82.1 (23)
**Renal Anemia** % (*n*)	25.0 (14)	21.4 (6)	28.6 (8)
**Hospital stay during treatment period** % (*n*)	12.5 (7)	11.1 (3)	15.4 (4)
**Hospital stay during follow-up period** % (*n*)	34.0 (19)	32.1 (9)	35.7 (10)
**Weight at baseline, kg** Mean (SD)	94.8 (12.5)	97.7 (12.4)	92.0 (12.2)
**BMI at baseline, kg/m**^**2**^ Mean (SD)	32.0 (3.0)	32.2 (3.0)	31.8 (3.0)
**Living in a partnership**, % (*n*)	64.3 (36)	57.1 (16)	71.4 (20)
**Employed**, % (*n*)	66.7 (36)	71.4 (20)	61.5 (16)
**IWQOL total** Mean (SD)	N = 52 83.0 (14.6)	N = 27 79.4 (17.8)	N = 25 86.9 (8.9)
**HADS anxiety** Mean (SD)	5.5 (4.0)	5.8 (4.1)	5.3 (4.0)
**HADS depression** Mean (SD)	5.1 (4.2)	5.7 (4.6)	4.4 (3.8)

HADS = Hospital Anxiety and Depression Scale, IWQOL-Lite = Impact of Weight on Quality of Life –Lite, SF-12 = Short Form 12, NODAT = New-onset diabetes after transplantation.

Standard maintenance immunosuppression consisted of a triple-drug regimen including a calcineurin inhibitor or m-TOR inhibitor, prednisolone (5 mg), and mycophenolate mofetil or azathioprine. Six patients received belatacept.

### 3.2. Weight related primary and longer-term outcomes

In the completer analysis, 32.0% (*n* = 8) of the patients in the IG and 16.7% (*n* = 4) of the patients in the CG achieved a weight loss of 5% or more. In the ITT analyzes (missing data coded as failure to achieve a 5% weight loss), the respective percentages were 28.6 and 14.3%. Even though the number of patients who achieved a weight reduction of 5% or more was twice as high in the IG compared to the CG, this difference was not statistically significant and the effect size was small (Completer: *χ*^2^ = 1.557, df = 1, *p* = 0.212; *φ* = 0.178; ITT: *χ*^2^ = 1.697, df = 1, *p* = 0.193; *φ* = 0.071; Table 3). Attainment of 5% weight loss did not significantly differ between groups across follow-up assessments but remained stable. Of the participants who completed the 12-month follow-up (*n* = 48), 45.8% lost further weight following treatment.

**Table 3:** Primary outcome.

Completer analysis			
Weight loss ≥5%	Intervention group	Control group	Statistics *X*^2^-Test
End of treatment % (*n*)	32.0 (8)*N* = 25	16.7 (4)*N* = 24	*X*^2^ = 1.557 (df = 1)*p* = 0.212, *φ* = 0.178
6-month follow-up % (*n*)	20.8 (5)*N* = 24	22.2 (6)*N* = 27	*X*^2^ = 0.014 (df = 1) *p* = 0.904, *φ* = 0.017
12-month follow-up % (*n*)	34.6 (9)*N* = 26	28.0 (7)*N* = 25	*X*^2^ = 0.259 (df = 1)*p* = 0.611, *φ* = 0.071
Intention-to-treat (ITT) analysis at end of treatment			
Weight loss ≥5%	Intervention group *N* = 28	Control group *N* = 28	Statistics *X*^2^-Test
End of treatment % (*n*)	28.6 (8)	14.3 (4)	*X*^2^ = 1.697 (df = 1) *p* = 0.193, φ = 0.174

At the end of treatment, the average percent weight lost in the completer sample and the ITT samples were 2.9% (SD 5.4) and 2.6% (SD 5.2) in the IG, and 0.6% (SD 4.2), and 0.5% (SD 3.9), respectively, in the CG. The differences between IG and CG were not statistically significant with Cohen’s d slightly below 0.5.

Achieving the weight loss goal of ≥5% was not associated with gender (female 18.5% vs. male 24.1%), insulin treatment (yes 18.2% vs. no 22.2%) time since transplantation (achieved weight loss goal: mean 77.6 months vs. did not achieve goal: mean 75.7 months), age at study entry (mean 49.2 vs. 47.6 years), and BMI at baseline (mean 32.3 vs. 31.9 kg/m^2^). None of the participants who required hospitalization during the treatment period achieved the weight loss goal.

**Figure 2:**
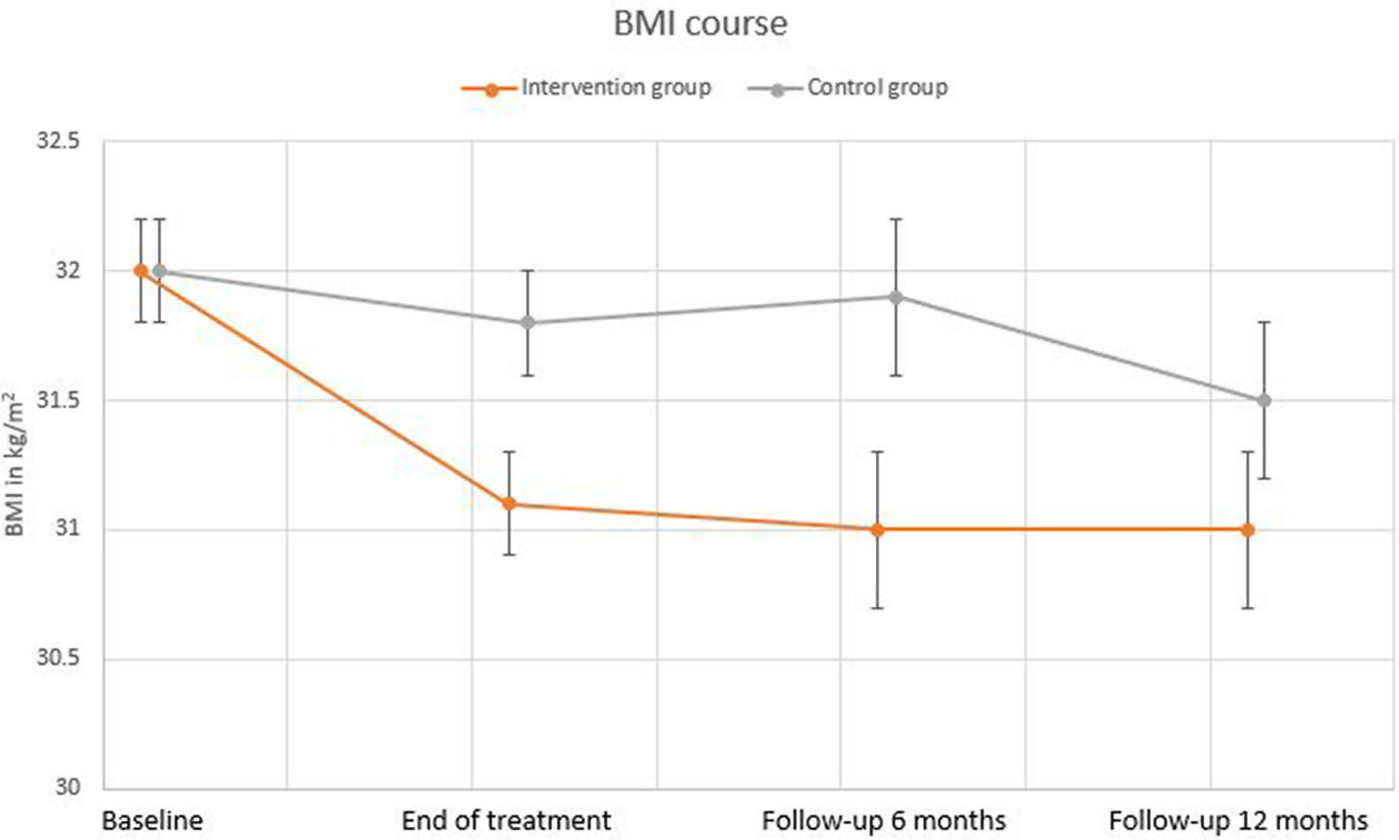
BMI course. BMI in kg/m^2^ at baseline, end of treatment, and 6 months and 12 months after the end of treatment in the intervention and control groups. LS means (SE) for baseline and end of treatment were derived from a mixed model for repeated measures (MMRM) including data from baseline and end of treatment only. LS means (SE) for the two follow-ups were derived from separate MMRMs including additional data up to 6-month and up to 12-month follow-up, respectively.

Considering the adjusted LS means from the 3 MMRMs, there was an overall significant reduction in BMI over time across groups (*F* = 2.977, df = 3, *p* = 0.034); however, the treatment-by-visit interaction effect was not statistically significant, neither at the end of treatment (*F* = 2.694, df = 1, *p* = 0.106) nor at the two follow-up time points (*F* = 2.431, df = 2, *p* = 0.093 and *F* = 1.458, df = 3, *p* = 0.228, respectively; Figure 2).

### 3.3. Secondary outcomes

Considering the adjusted LS means, there was an overall significant increase in eGFR over time (*F* = 3.070, df = 3, *p* = 0.030) but no treatment-by-visit interaction (*F* = 1.994, df = 3, *p* = 0.118).

**Figure 3:**
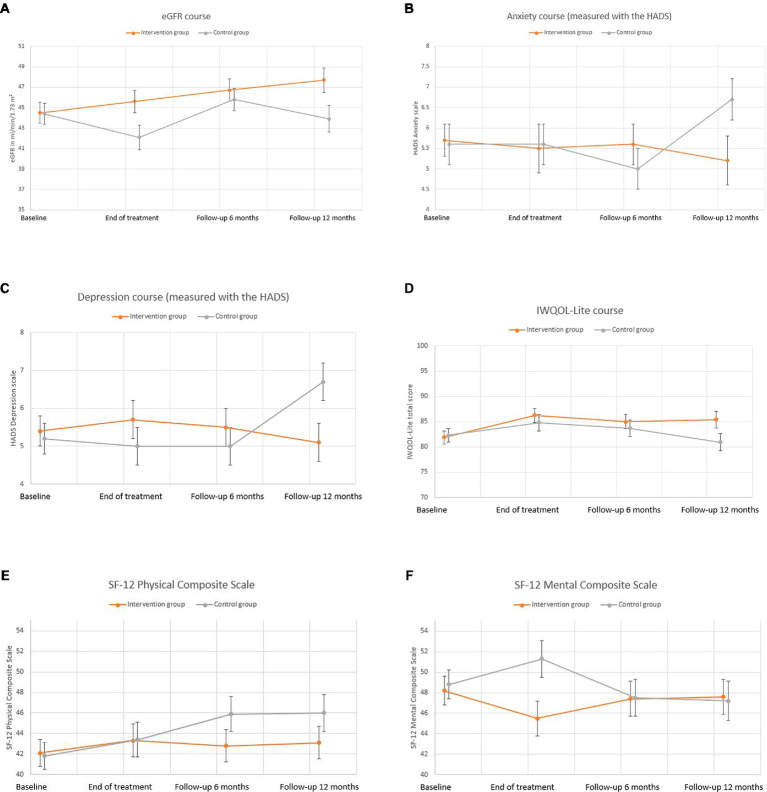
**(A)** eGFR course. **(B)** Anxiety course. **(C)** Depression course. **(D)** IWQOL-Lite course. **(E)** SF-12 Physical Composite Scale. **(F)** SF-12 Mental Composite Scale. **(A–F)** Secondary outcome measures at baseline, end of treatment, and 6 months and 12 months after the end of treatment in the intervention and control groups. LS means (SE) were derived from a mixed model for repeated measures (MMRM) including all data from baseline up to 12-month follow-up. BMI=Body Mass Index, eGFR = estimated glomerular filtration rate, HADS=Hospital Anxiety and Depression Scale, IWQOL-Lite = Impact of Weight on Quality of Life-Lite, SF-12 = Short Form 12.

With regard to quality of life (IWQOL-Lite, SF-12) and levels of anxiety and depression (HADS), the mean values were mostly within normal range and did not show any significant group differences over time. However, the IWQOL-Lite total score increased significantly over time across samples (*F* = 3.311, df = 3, *p* = 0.023; Figures 3A–F; Supplementary Table S2).

### 3.4. Adverse events and unplanned hospital admissions

There were no adverse events attributed to trial participation. One patient with type II diabetes and cardiovascular disease who was randomized to the CG deceased during the last 6 months of the follow-up period 31 months after transplantation. One patient in the IG experienced a heart attack a few days after the baseline visit and required hospitalization. The patient was able to continue study participation.

There was a significant number of unplanned hospital admissions among participants. During the treatment phase, 4 patients (15.4%) in the CG and 3 patients (11.1%) in the IG were admitted to the hospital. Overall, the median stay was 6 days (range 1–41). Most of these admissions had a minimal impact on study participation; however, one patient in the CG required a hospital stay of 41 days due to a cardiovascular event and dropped out of the study. During follow-up, 10 patients (35.7%) in the CG and 9 (32.1%) in the IG required hospitalization. The most frequent cause for hospitalizations were urinary tract infections; however, during the follow-up period, two patients were admitted due to COVID-19 infection and were hospitalized for 7 (IG) and 25 (CG) days, respectively.

## 4. Discussion

To our knowledge, this study is one of the rare attempts to support weight loss in KTx recipients with overweight and obesity. The primary study objective of 55% of the participants in the IG attaining a ≥ 5% weight loss compared to 15% in the CG was not reached and there was no statistical significant difference between groups. The percentage of patients reaching the 5% weight loss goal in the IG was nearly twice as high compared to the CG with a small effect size (32% versus 16.7%; *φ* = 0.178). It is quite astonishing that the number of patients in the CG who achieved at least 5% weight loss in our study is almost identical to the results of a large RCT involving patients with obesity in primary care (40). In this study, 16% of the 367 patients who were randomized to 10TT achieved at least 5% weight loss at 3 months and 27% at 24 months. The weight loss with the 10TT leaflet is notable for a minimal intervention that seems to also be effective in modifying the behavior in this special population of KTx patients.

There was an overall significant reduction in BMI over time across groups without a statistically significant difference between groups. The overall weight loss at the end of treatment was modest with 2.9% (SD 5.4) in the IG and 0.6% (SD 4.2) in the CG. However, mean weight loss was largely maintained at 12-month follow-up. This is promising given that most weight loss studies see weight regain post-treatment. In addition, the participants were severely ill, with a low average transplant function and high rates of severe comorbidities with 40% requiring hospitalization at some point during the treatment and follow-up periods. However, interventions focusing on habitual behaviors might have the potential to break old habits and form new habits which might support weight-loss maintenance.

Most RCTs aiming at preventing weight gain early after KTx did not demonstrate any advantage of either nutritional or physical activity-based interventions over standard care (24, 25). There are several trials on weight-loss interventions in patients with chronic physical or mental illnesses, e.g., rheumatoid arthritis, breast cancer, mental disorders, and cardiovascular diseases (44–47). Treating these patients can be challenging, as general recommendations often need adaption to meet specific demands associated with the comorbidities. Consequently, it is of great importance to put effort in the adjustment of the intervention for the specific patient group to be able to achieve clinically meaningful results.

Kidney function (eGFR) significantly increased across groups. Even though tempting to speculate, this increase might not be associated with weight loss but rather with the optimization of treatment, e.g., during hospitalizations.

Quality of life and levels of depression and anxiety were generally within normal range and did not change over time with the exception of a significant improvement of obesity-related quality of life across groups.

Treatment adherence was high. Nevertheless, dropout rates during the trial were 10.7% (*n* = 3) in the CG and 7.1% (*n* = 2) in the IG. There was only one study dropout in the IG during the treatment phase which occurred due to severe orthopedic complications not associated to the study, which made a treatment continuation impossible. Apart from that, nearly all sessions were completed by the remaining participants. It is important to highlight that all participants were highly motivated to participate in this trial. This limits the transferability of the results to the whole group of KTx patients. As KTx patients can be confronted with a multitude of potential complications and medical difficulties, participating in a weight loss intervention might be of subordinate importance for a considerable proportion of KTx patients. It seems crucial to better inform KTx patients on the association between obesity and adverse post-transplant outcomes.

The reasonable high rate of hospitalization was not necessarily unexpected. Interestingly, none of these patients reached the weight loss goal during the treatment phase and, thus, did not appear to have lost weight due to the underlying reasons for their hospital admissions during the intervention period.

### 4.1. The putative influence of the COVID pandemic

This clinical trial was ongoing at the onset of the COVID-19 pandemic which might have significantly influenced study conduct in several ways. Most of the trial took place during the COVID-19 stay-at-home order and transplant patients were advised to only visit the transplant outpatient clinic in case of severe difficulties. KTx patients were considered to be at risk and were strongly advised to self-isolate and refrain from any activity which might be associated with an increased risk to get infected with SARS-CoV-2.

The study design included the option for telemedicine-based interventions and in the first pandemic year 2020, all participants in the IG relied on this option. Since the CG was self-guided with only one initiation session, the influence on study conduct was less profound. Implementing telehealth-delivered approaches has the potential to circumnavigate the challenges of intervention delivery while social distancing measures are in place and consequently might improve adherence rates and reduce attrition. There is evidence that telemedicine-based programs for KTx patient care support the maintenance of physical activity also during COVID-19 restrictions (48). However, literature has also shown that a surprisingly large proportion of patients with chronic kidney disease in Germany, especially older patients and patients with a lower educational level, do not use the Internet at all and that the majority of Internet users reported that they have not used Internet-based technologies within a medical context so far (49). Consequently, since we did not provide the participants with a tablet computer, a large proportion of sessions during the lockdown were conducted over the phone. However, the study of Nguyen et al. (50) suggests that video conferencing might be superior to telephone-based consultations.

During the COVID-19 pandemic, stay-at-home orders and social distancing disrupted daily routines and impacted health behaviors, including physical activity and eating habits. Global estimates suggest that 12.8–48.6% of community-dwelling adults reported weight gain associated with lifestyle changes during the COVID-19 pandemic, with higher odds of weight gain among those with elevated baseline BMI (51). Apart from that, daily life was severely affected by a variety of regulations leading, e.g., to a temporary closure of sports and leisure facilities and an increased percentage of people working from home. These circumstances seemed to be associated with an increase in sedentary behavior and a decrease of PA (52). Thus, our cognitive-behavioral intervention efforts may have been impeded by influences of the COVID-19 pandemic on daily and instrumental activities (e.g., childcare, healthcare access), stress, and health behaviors (i.e., physical activity, diet, sleep).

Investigating the impact of the COVID-19 pandemic on the effectiveness of cognitive-behavioral interventions is important as this may help tailoring of interventions to the different needs of people among this high-risk population during the still ongoing pandemic (53).

Finally, given the restrictions to clinical visits to the hospital, also study assessments could not be conducted face-to-face. As the patients were not equipped with calibrated scales, participants were asked to send the body weight and blood test results measured at their local nephrologist’s office at the next opportunity to keep deviations as small as possible. Measurement of body composition was planned but could not be reliably executed outside of the transplant center. Additionally, the self-rating instruments were sent to the participants by mail which might have led to more missing values than we would expect when patients are allowed to regularly visit the transplant center.

### 4.2. Strengths and weaknesses

We note potential strengths and weaknesses. Strengths include the RCT design, testing scalable interventions of relevance to patients after KTx living far away from the transplant center, and high retention rates through 12 months of follow-up. In contrast to the majority of weight loss interventions, in which the participants are predominantly female, there was a balanced sex ratio in our trial. Weaknesses include the relatively small sample size which represents a potential limitation in terms of statistical power to detect small differences. Apart from that, in this pilot study we refrained from monitoring laboratory parameters including plasma lipid, blood glucose levels, and clinical measurements such as blood pressure associated with obesity and metabolic syndrome. In future multicenter trials, these parameters should be included as secondary outcomes and specifically addressed. All participants were highly motivated to participate in this study. Therefore, they represent a special group which is probably not representative for the entirety of KTx recipients. We note that the findings may not generalize to different transplant centers or to different clinicians for delivering the intervention. Finally, we did not systematically investigate the impact of the COVID-19 pandemic on the individual. In future studies, longer and more intensive interventions may be needed to provide greater weight loss benefit to patients after KTx. The mainly cognitive-behaviorally oriented treatment might be improved by adding an exercise component and increasing the nutritional component. Additionally, the effects of a moderate weight loss on graft function, levels of the immunosuppressive medication, post-transplant morbidity, and mortality need to be investigated in large scale long-term studies.

## 5. Conclusion

Short-term, telehealth-delivered, and cognitive-behaviorally oriented scalable weight loss treatment seems to be feasible and acceptable for patients after KTX who suffer from overweight or obesity. The primary objective has not been reached, as IG and CG did not differ significantly with regard to the weight loss goal of ≥5% (32% versus 17%). It has to be kept in mind that the COVID-19 pandemic and restrictions associated to it might have exerted a negative influence on the study conduct and also on the health behaviors of the participants that may have counteracted our intervention efforts.

Longer and more intensive interventions may be needed to provide greater weight loss benefit to patients after KTx. Adding an exercise component and increasing the nutritional component might outperform the mainly cognitive-behaviorally oriented treatment. However, they need to be safe for this vulnerable group and allow the participation also of patients with severe comorbidities.

## Data availability statement

The raw data supporting the conclusions of this article will be made available by the authors, without undue reservation.

## Ethics statement

The studies involving human participants were reviewed and approved by the Institutional Ethics Review Board of Hannover Medical School. The patients/participants provided their written informed consent to participate in this study.

## Author contributions

MN and MZ designed the trial with support from FG and ES. BJ provided content from the “Lighter through Life” program. MN obtained research funding. DCB was essential in the recruitment process of the study and conducted baseline and follow-up visits. MN, R-KV, and KL performed cognitive-behavioral interventions. ES was responsible for the nutritional counseling. FG and GE provided nephrological support. DCB was responsible for data input. MZ, MN, and DCB analyzed the data. MZ and MN wrote the first draft. All authors contributed to the article and approved the submitted version.

## Funding

The study was supported by a grant to Mariel Nöhre sponsored by the Ministry for Science and Culture of Lower Saxony as project of the “Center for Organ Regeneration and Replacement (CORE),” Transplant Center, Hannover Medical School. This work was supported in part by the Ellen Schmidt Habilitation Program of Hannover Medical School for Mariel Nöhre.

## Conflict of interest

The authors declare that the research was conducted in the absence of any commercial or financial relationships that could be construed as a potential conflict of interest.

## Publisher’s note

All claims expressed in this article are solely those of the authors and do not necessarily represent those of their affiliated organizations, or those of the publisher, the editors and the reviewers. Any product that may be evaluated in this article, or claim that may be made by its manufacturer, is not guaranteed or endorsed by the publisher.
