# Supplementary material for: Cognitive-behavioral and dietary weight loss intervention in adult kidney transplant recipients with overweight and obesity: Results of a pilot RCT study (Adi-KTx)
Source: Front Psychiatry. 2023 Apr 11;14:1071705. doi: 10.3389/fpsyt.2023.1071705 (PMC10126341; doi:10.3389/fpsyt.2023.1071705)
Supplement: Supplementary file 1 [file Table_1.DOCX]

Supplementary Material

# Supplementary Tables

Supplementary Table 1: Comparison of characteristics between groups

|  | **Total** | **Intervention group (IG)** | **Control group (CG)** | **Statistics**  **(Mann-Whitney-U-tests and Chi-Square-tests)**  **IG vs. CG** |
| --- | --- | --- | --- | --- |
| **N** | **56** | **28** | **28** |  |
| **Age at baseline, years**  Mean (SD) | 48.0 (12.3) | 48.2 (11.4) | 47.7 (13.3) | Z=-0.147, p=0.883 |
| **Sex, women** % (n) | 48.2 (27) | 42.9 (12) | 53.6 (15) | X^2^=0.644 (df=1) p=0.422 |
| **Educational level**, ≥12 years of formal education, % (n) | 26.8 (15) | 28.6 (8) | 25.0 (7) | X^2^=0.091 (df=1) p=0.763 |
| **eGFR ml/min/1.73 m²**  Mean (SD) | 44.9 (15.8) | 40.5 (14.1) | 49.4 (16.5) | Z=-1.697, p=0.090 |
| **Time since KTx, months**  Mean (SD) | 77.4 (68.4) | 67.8 (69.8) | 87.1 (66.9) | Z=-1.475, p=0.140 |
| **Dialysis before KTx**, % (n) | 80.4 (45) | 89.3 (25) | 71.4 (20) | **X^2^=4.139 (df=1)** **p=0.042** |
| **Living kidney donation**, % (n) | 33.9 (19) | 32.1 (9) | 35.7 (10) | X^2^=0.080 (df=1) p=0.778 |
| **Diabetes mellitus,** % (n)  type 1, n  type 2, n  NODAT, n  Insulin therapy, n | 28.6 (16)  5  6  5  11 | 28.6 (8)  2  4  2  6 | 28.6 (8)  3  2  3  5 | X^2^=0.000 (df=1) p=0.999 |
| **Coronary Heart Disease** % (n) | 8.9 (5) | 10.7 (3) | 7.1 (2) | X^2^=0.220 (df=1) p=0.639 |
| **Hypertension**  % (n) | 83.9 (47) | 85.7 (24) | 82.1 (23) | X^2^=0.132 (df=1) p=0.716 |
| **Renal Anemia** % (n**)** | 25.0 (14) | 21.4 (6) | 28.6 (8) | X^2^=0.381 (df=1) p=0.537 |
| **Hospital stay during treatment period** % (n) | 12.5 (7) | 11.1 (3) | 15.4 (4) | X^2^=0.163 (df=1) p=0.686 |
| **Hospital stay during follow-up period** % (n) | 34.0 (19) | 32.1 (9) | 35.7 (10) | X^2^=0.080 (df=1) p=0.778 |
| **Weight at baseline, kg**  Mean (SD) | 94.8 (12.5) | 97.7 (12.4) | 92.0 (12.2) | Z=-1.688, p=0.091 |
| **BMI at baseline, kg/m^2^**  Mean (SD) | 32.0 (3.0) | 32.2 (3.0) | 31.8 (3.0) | Z=-0.508, p=0.661 |
| **Living in a partnership**, % (n) | 64.3 (36) | 57.1 (16) | 71.4 (20) | X^2^=1.244 (df=1) p=0.265 |
| **Employed**, % (n) | 66.7 (36) | 71.4 (20) | 61.5 (16) | X^2^=1.244 (df=1) p=0.265 |
| **IWQOL total**  Mean (SD) | **N=52**  83.0 (14.6) | **N=27**  79.4 (17.8) | **N=25**  86.9 (8.9) | Z=-1.237, p=0.216 |
| **HADS anxiety**  Mean (SD)  **HADS depression**  Mean (SD) | 5.5 (4.0)  5.1 (4.2) | 5.8 (4.1)  5.7 (4.6) | 5.3 (4.0)  4.4 (3.8) | Z=-0.337, p=0.736  Z=-1.153, p=0.249 |

HADS=Hospital Anxiety and Depression Scale, IWQOL-lite=Impact of Weight on Quality of Life –Lite, SF-12=Short Form 12, NODAT= New-onset diabetes after transplantation

**Supplementary Table 2: LS means and 95% confidence intervals of primary and secondary outcome variables over time**

|  | Baseline | | End of treatment | | 6-month follow-up | | 12-month follow-up | | Statistics | |
| --- | --- | --- | --- | --- | --- | --- | --- | --- | --- | --- |
| Outcome variables | Intervention | Control | Intervention | Control | Intervention | Control | Intervention | Control | over time | treatment-by-visit interaction |
|  | LS mean (95% CI) | LS mean (95% CI) | LS mean (95% CI) | LS mean (95% CI) | LS means (95% CI) | LS means (95% CI) | LS means (95% CI) | LS means (95% CI) |  |  |
| BMI, kg/m^2^ | 32.0  (31.5; 32.4) | 32.0  (31.5; 32.4) | 31.1  (30.6; 31.6) | 31.8  (31.4; 32.3) | 31.0  (30.5: 31.6) | 31.9  (31.3; 32.4) | 31.0  (30.3; 31.6) | 31.5  (30.8; 32.1) | F=2.977, df=3, p=.034 | ns |
| eGFR (ml/min/1.73m^2^) | 44.5  (42.4; 46.5) | 44.4  (42.4; 46.5) | 45.6  (43.4; 47.8) | 42.1  (39.8; 44.4) | 46.7  (44.6; 48.9) | 45.8  (43.7; 48.0) | 47.7  (45.4; 50.1) | 43.9  (41.5; 46.4) | F=3.070, df=3, p=.030 | ns |
| Anxiety (HADS) score | 5.7  (4.8; 6.5) | 5.6  (4.7; 6.5) | 5.5.  (4.4; 6.6) | 5.6  (4.5; 6.6) | 5.6  (4.6; 6.6) | 5.0  (3.9; 6.0) | 5.2  (4.1; 6.3) | 6.7  (5.6; 7.8) | ns | ns |
| Depression (HADS) score | 5.4  (4.6; 6.2) | 5.2  (4.4; 5.9) | 5.7  (4.7; 6.7) | 5.0  (4.1; 6.0) | 5.5  (4.7; 6.4) | 5.0  (4.1; 6.0) | 5.1  (4.1; 6.1) | 6.7  (5.7; 7.6) | ns | ns |
| IWQOL-Lite total score (standardized) | 81.9  (79.4; 84.5) | 82.3  (79.7; 84.9) | 86.3  (83.5; 89.0) | 84.8  (81.6; 87.9) | 85.0  (82.2; 87.9) | 83.7  (80.6; 86.8) | 85.4  (82.2; 88.6) | 80.9  (77.6; 84.2) | F=3.311, df=3, p=.023 | ns |
| SF-12 Physical Composite Scale | 42.1  (39.5; 44.6) | 41.8  (39.2; 44.4) | 43.3  (40.2; 46.4) | 43.4  (40.1; 46.7) | 42.8  (39.7; 45.9) | 45.9  (42.6; 49.2) | 43.1  (40.0; 46.3) | 46.0  (42.5; 49.5) | ns | ns |
| SF-12 Mental Composite Scale | 48.2  (45.4; 51.0) | 48.8  (45.9: 51.6) | 45.5  42.2; 48.9) | 51.3  (47.7; 54.8) | 47.4  (44.1; 50.8) | 47.5  (43.9; 51.0) | 47.6  (44.1; 51.0) | 47.2  (43.4; 50.9) | ns | ns |

BMI=Body Mass Index, eGFR=estimated glomerular filtration rate, HADS=Hospital Anxiety and Depression Scale, IWQOL-lite=Impact of Weight on Quality of Life –Lite, SF-12=Short Form 12, ns = not significant

LS means are based on MMRMs adjusted for the respective baseline values. Data are also depicted in Figures 3a-f.
